# Integrating genome‐wide association mapping of additive and dominance genetic effects to improve genomic prediction accuracy in *Eucalyptus*

**DOI:** 10.1002/tpg2.20208

**Published:** 2022-04-20

**Authors:** Biyue Tan, Pär K. Ingvarsson

**Affiliations:** ^1^ Umeå Plant Science Centre, Dep. of Ecology and Environmental Science Umeå Univ. Umeå SE‐90187 Sweden; ^2^ Stora Enso AB Nacka SE‐131 04 Sweden; ^3^ Linnean Centre for Plant Biology, Dep. of Plant Biology, Uppsala BioCenter Swedish Univ. of Agricultural Sciences Uppsala SE‐750 07 Sweden

## Abstract

Genome‐wide association studies (GWAS) is a powerful and widely used approach to decipher the genetic control of complex traits. Still, a significant challenge for dissecting quantitative traits in forest trees is statistical power. This study uses a population consisting of 1,123 samples derived from two successive generations of crosses between *Eucalyptus grandis* (W. Hill) and *E. urophylla* (S.T. Blake). All samples have been phenotyped for growth and wood property traits and genotyped using the EuChip60K chip, yielding 37,832 informative single nucleotide polymorphisms (SNPs). We use multi‐locus GWAS models to assess additive and dominance effects to identify markers associated with growth and wood property traits in the eucalypt hybrids. Additive and dominance association models identified 78 and 82 significant SNPs across all traits, respectively, which captured between 39 and 86% of the genomic‐based heritability. We also used SNPs identified from the GWAS and SNPs using less stringent significance thresholds to evaluate predictive abilities in a genomic selection framework. Genomic selection models based on the top 1% SNPs captured a substantially greater proportion of the genetic variance of traits compared with when we used all SNPs for model training. The prediction ability of estimated breeding values improved significantly for all traits when using either the top 1% SNPs or SNPs identified using a relaxed *p* value threshold (*p *< 10^–3^). This study also highlights the added value of incorporating dominance effects for identifying genomic regions controlling growth traits in trees. Moreover, integrating GWAS results into genomic selection method provides enhanced power relative to discrete associations for identifying genomic variation potentially valuable for forest tree breeding.

## Core Ideas

- Multi‐locus genome‐wide association studies (GWAS) models were used to assess both additive and dominance effects.
- Single nucleotide polymorphisms (SNPs) identified from the GWAS were used in genomic selection.
- Filtering SNPs based on GWAS results increase predictive ability in genomic selection.
- Both additive and dominance effects are important in interspecific hybrids.

**Abbreviations**
CBH: circumference at breast height
CBH3: circumference at breast height at age 3 yr
CBH6: circumference at breast height at age 6 yr
CV: cross‐validation
FarmCPU: fixed and random model circulating probability unification
GS: genomic selection
GWAS: genome‐wide association studies
Ht3: height at 3 yr old
Ht6: height at 6 yr old
QQ: quantile‐quantile
QTLs: quantitative trait loci
PCA: principal component analysis
SNPs: single nucleotide polymorphisms.

## 1 INTRODUCTION

Deciphering the genetic basis of complex phenotypic traits is of fundamental importance for understanding biological processes and may ultimately provide information that can help enhance selection in plant breeding programs. Genome‐wide association studies (GWAS) are a powerful way to identify putative causal genes or genomic segments that underlie plant phenotypic variation, particularly for traits with complex genetic architectures (Ingvarsson & Street, 2011; Kruglyak, 2008). Dissection of complex traits has been undertaken in forest genetics to understand the genetic basis of adaptive phenotypes (Ingvarsson et al., 2008; Olson et al., 2013; Wang et al., 2018) and physiological or morphological traits, such as growth or wood properties. For example, Porth et al. (2013) and later Chhetri et al. (2019) performed GWAS for wood traits, biomass, eco‐physiological and phenology traits in *Populus trichocarpa* L. with genotyping based on 6.78 million single nucleotide polymorphisms (SNPs). Similarly, a study of *Salix viminalis* L. identified 29 SNPs that were associated with bud burst, leaf senescence, number of shoots, or shoot diameter (Hallingback et al., 2016). In *Eucalyptus*, the earliest GWAS identified 16 markers associated with growth and two associated with lignin traits (Cappa et al., 2013). Recently, 26 quantitative trait loci (QTLs) were identified for productivity and disease resistance using a regional heritability mapping method that helps increase the genomic heritability explained to 5–15% from 4–6% when using individual SNPs (Resende et al., 2017a; Resende et al., 2017b).

GWAS studies can also provide tools for accelerating the long breeding cycles in forest tree breeding (reviewed in Neale & Kremer, 2011). For example, although many species of *Eucalyptus* display unusually fast growth, breeding cycles aimed at developing elite commercial genotypes still take between 12 to 16 years to complete since the identification of elite genotypes requires progeny trials followed by two or more sequential clonal trials (Rezende et al., 2014). However, genomic selection based on genome‐wide molecular makers is expected to reduce the time required for completing a cycle of developing elite clones to only nine years. This reduction comes mainly from the shorter time needed for progeny tests when phenotypes can be predicted from the genomic selection models (Grattapaglia, 2017).

**Core Ideas**

- Multi‐locus genome‐wide association studies (GWAS) models were used to assess both additive and dominance effects.
- Single nucleotide polymorphisms (SNPs) identified from the GWAS were used in genomic selection.
- Filtering SNPs based on GWAS results increase predictive ability in genomic selection.
- Both additive and dominance effects are important in interspecific hybrids.

The rapid development in genomics has opened up opportunities to identify molecular markers associated with traits of interest and use these marker–trait associations to complement and extend traditional breeding programs. Despite the efforts to discover polymorphisms associated with economically relevant traits, much of the genetic contribution to complex traits in forest trees remains unexplained. One of the main reasons is that GWAS methods conduct tests on markers individually, using generalized linear models or mixed linear models. When dealing with complex traits such as growth and wood qualities, where the effect size of individual loci is likely small to moderate, these methods suffer from limited statistical power to detect loci of small effects (Müller et al., 2017). One potential approach to increase the power and accurately identify more causal variants is the so‐called multi‐locus mixed model. Multi‐locus mixed models simultaneously test multiple markers by including them as covariates in a stepwise mixed linear model to partially remove confounding between tested markers and kinship (Segura et al., 2012). One such method is the fixed and random model circulating probability unification (FarmCPU) that performs marker tests using other associated markers as covariates in a fixed‐effect model (Liu et al., 2016). The FarmCPU then optimizes the associated covariate markers separately using a random effect model. This approach simultaneously reduces computational complexity; removes confounding between population structure, kinship, and QTL; prevents model over‐fitting; and controls the number of false positives (Liu et al., 2016).

To date, most GWAS analyses have been undertaken by implicitly assuming a genetic architecture consisting of additive effects. However, non‐additive effects, including dominance (Bruce, 1910), over‐dominance (Crow, 1948) and epistasis (Hill, 1982), also play important roles in controlling some traits. One trait where non‐additive effects are likely to be pronounced is heterosis, or hybrid vigor is the near‐universally observed phenomenon of phenotypic superiority of hybrid progeny relative to their parents (Charlesworth & Willis, 2009). Not surprisingly, heterosis has been and continues to be of great importance in most plant breeding schemes (Duvick, 2001). To date, a limited number of studies have utilized GWAS methods to dissect the genetic basis of heterotic traits in *Arabidopsis thaliana* (L.) and rice (*Oryza* L.). In the model plant *A. thaliana*, dominance and over‐dominance of flowering time are well‐studied traits, and significant loci from a GWAS explain as much as 20% of the phenotypic variation in a hybrid population consisting of 435 individuals derived from inter‐crossing 30 parents (Seymour et al., 2016). In rice, genome‐wide dissection uncovered multiple non‐additive effect loci for yield increase (Li et al., 2016; Zhen et al., 2017), for example, rice heterosis 8 (*RH8*), a major QTL regulating grain‐yield component traits (Li et al., 2016). In *Eucalyptus* hybrids, dominance appears to be an important and widespread contributor to many growth‐related traits (Bison et al., 2006; Bouvet & Vigneron, 1995; Volker et al., 2008), and ratios of dominance to additive variances exceeding 1.2 have been estimated for growth in *E. grandis* × *E. urophylla* hybrids (Bouvet et al., 2009; Makouanzi et al., 2014; Tan et al., 2017). Such results suggest that there should be ample opportunities to identify SNPs accounting for dominance or over‐dominance effects in *Eucalyptus* hybrids.

In recent years, another genomic‐based approach widely used in plant and animal breeding is genomic selection (GS), alternatively referred to as genomic prediction. Unlike GWAS, GS refers to marker‐based selection where total genetic variance is captured using genome‐wide markers without a prior step of identifying trait‐associated markers. Genomic selection aims to predict the genetic potential (e.g., genome‐estimated breeding values) of breeding individuals without locating genes or QTLs important for the trait(s) of interest. One of the most critical questions for GS is how to improve the prediction accuracy, and methods for accuracy has long been a central research aim in genomic selection. Thus far, progress on increasing prediction accuracy has been achieved by developing new statistical models, more efficient design of training populations, improved quality of phenotypic measurements, a greater number of makers used for model building, and considering non‐additive effects (Grattapaglia, 2017). In this paper, we assess methods for improving genomic prediction accuracy by integrating results from GWAS studies into GS to predict the genetic potential of breeding targets. Using only associated SNPs identified from a GWAS is usually not sufficient for explaining a significant fraction of the genetic variation in a trait of interest (the so‐called “missing heritability” problem; Makowsky et al., 2011). However, utilizing GWAS information in the form of associated SNPs with other types of data can enhance prediction ability in GS studies (Gowda et al., 2015).

In this study, we present results from a GWAS on growth and wood quality traits in a breeding population comprising two species of *Eucalyptus, E. grandis* (W. Hill) and *E. urophylla* (S.T. Blake), and their hybrids. We also integrate the GWAS results in a GS model to assess whether this help increases prediction accuracies for the traits in question. Specifically, our study has two objectives: first, we implement a state‐of‐the‐art GWAS method that considers additive and dominance effects to dissect the genetic architecture of growth and wood quality traits. We also evaluate the proportion of phenotypic variation explained by significant loci for these two genetic effects. Second, we evaluate how different categories of informative SNPs, selected based on the results from a GWAS, can be implemented in genomic prediction to estimate variance components and to evaluate prediction accuracies of estimated breeding values.

## 2 RESULTS

### 2.1 Growth and wood traits

All growth traits were moderately variable at the different assessment ages (Table 1). We observed a lower phenotypic variation for height at 3 yr of age, as judged by the coefficient of variation (Table 1). The F1 population underwent selection based on height to identify trees for genotyping. This selection process likely contributed to the lower phenotypic variation we see in height at 3 yr. We also observed low phenotypic variation for basic density and pulp yield, commonly observed in many wood quality traits. Generally, variation in circumference at breast height (CBH) was greater than in height, but both mean and variance for both traits increased as the trees aged. Growth traits generally had low heritabilities (*h* ^2^ < 0.2), whereas wood quality traits showed moderate heritabilities (Table 1). Phenotypic correlations between growth traits were generally positive (0.24∼0.74), whereas basic density was weakly negatively correlated with pulp yield (−0.28). The wood quality traits were generally independent of growth traits (correlations in the range −0.1 to 0.1) (Figure S1). The greatest positive phenotypic correlations were observed between CBH and height assessed at the same age (0.63 and 0.74 for 3 and 6 yr, respectively).

**TABLE 1:** Statistical summary of phenotypes

Trait	Abbr.	No. obs.	Unit	Mean	Coeff. Var.	*h* ^2^
					**%**	
Circumference at breast height, age 3 yr	CBH3	1,123	cm	61.82	13.22	0.143
Height, age 3 yr	Ht3	1,094	m	22.43	9.81	0.162
Circumference at breast height, age 6 yr	CBH6	1,104	cm	83.80	18.67	0.186
Height, age 6 yr	Ht6	985	m	28.40	13.09	0.182
Basic density	BD	1,061	kg m^3^	532.78	6.83	0.381
Pulp yield	PY	1,039	%	49.64	8.05	0.42

*Note*. Abbr., abbreviation; Coeff. Var, coefficient of variance; No. obs., number of observations.

### 2.2 Population structure and model optimization

**FIGURE 1:**
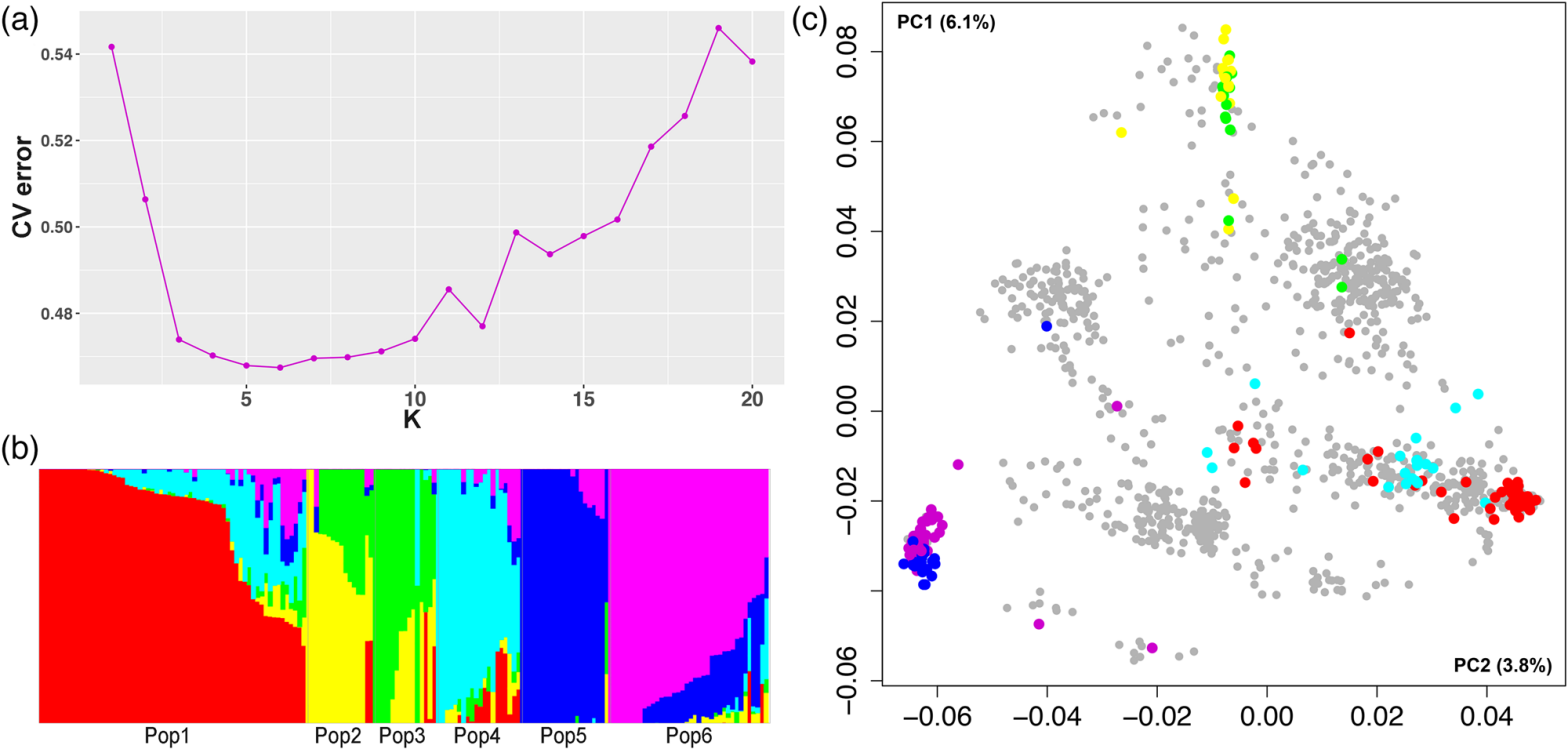
Population structure of parents and F1 progenies. A: Cross‐validation (CV) error in the admixture analysis for K varying from 1 to 20 for the 174 parents. B: Population structure of parents inferred using admixture for K = 6. C: PCA plot based on genetic covariance among all individuals. Only the first two principle components are shown. The colors used for the parents are in line with the clustering shown in panel B, with grey color denoting all F1 progeny

To examine population structure in the breeding population, including parents and their F1 progenies, we conducted model‐based admixture and fastStructure analyses and principal component analysis (PCA) based on a set of independent SNPs. The admixture analysis could not identify an optimal genetic clustering by minimizing the cross‐validation (CV) error even for K‐values up to K = 100 (Figure S2). In contrast, fastStructure suggested an optimal genetic clustering of K = 1. Because of the inconsistencies between the methods, we repeated the population structure analyses using only the parents, given that the F1 individuals were all obtained through crossings between these parents. Admixture analyses based on the parents alone suggested K = 6 minimized the CV error (Figure 1a), and K = 6 was also the optimal genetic clustering obtained from fastStructure. The parents were assigned to the six subpopulations according to individual ancestry proportions (Figure 1b). We also performed a PCA to summarize genetic variation among parents, and the first six components explained 21.53% of the total genetic variation. Notably, the eigenvalues beyond the first six PCs were relatively small (Figure S3), consistent with the minimum K identified in the admixture analyses. Parents are separated into three clusters based on the first two principal components, and two further sub‐clusters occur within each major cluster. Progenies are derived from crossing parents either with the different major clusters or between them (Figure 1C) and therefore, we used the first six PCs in all subsequent analyses to correct for population stratification.

### 2.3 GWAS for additive effects

**FIGURE 2:**
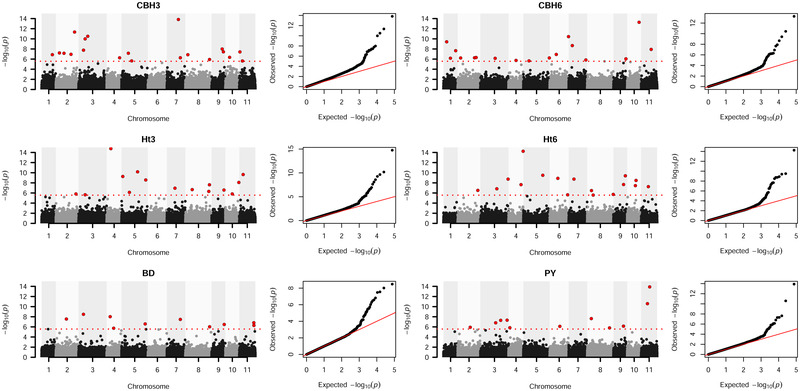
Manhattan plots and quantile‐quantile (QQ) plots of the fixed and random model circulating probability unification (FarmCPU) results using an additive effects model. The traits used are CBH and height at age 3 and 6 (Ht3 and Ht6, respectively) as well as basic density (BD) and pulp yield (PY). The Manhattan plots show –log_10_ *p* values plotted against SNP positions on the 11 *Eucalyptus* chromosomes. Associations reaching genome‐wide significance are displayed in red and the horizontal dotted line indicates a Bonferroni‐corrected significant threshold of *p* < 1.7 × 10^–6^. The QQ plots for each of the six traits demonstrate the observed versus expected distribution of *p* values. The solid red line represents the expected null distribution assuming no associations. BD, basic density; CBH3, circumference at breast height at age 3 yr; CBH6, circumference at breast height at age 6 yr; Ht3, height at age 3 yr; Ht6, height at age 6 yr; PY, pulp yield

We first ran FarmCPU with an additive effect encoding to identify loci with significant additive effects on the different phenotypes. Quantile‐quantile (QQ) plots suggest that population structure and kinship relationships were well controlled in the GWAS for the different traits (Figure 2). SNPs with *p* values < 1.7 × 10^–6^ threshold were declared statistically significant. Overall, we identified 78 significant SNPs across the six traits, and these significant SNPs were distributed across all 11 chromosomes (Figure 2). Both CBH and height show strong genetic correlations across ages and a few significant SNPs were also shared across these traits (none for additive effects and one between circumference at breast height at age 3 yr (CBH3)–height at 3 yr old (Ht3) and two between circumference at breast height at age 6 yr (CBH6)–height at 6 yr old (Ht6) for dominance effects, Table S3). Even when extending the distance between possible significant SNPs to 100 kb, the number overlapping associations remain few (one for additive effects and four for dominance effects; Table S3). Growth traits had more significant SNPs than wood traits, with height and CBH at the two different ages having between 14 and 18 significant SNPs, whereas only nine significant SNPs were identified for the two wood quality traits. We generally observe lower phenotypic variances explained by individual SNPs for CBH and height than pulp yield and basic density (Table S1). The maximum percentage of phenotypic variance explained by a single associated SNP was 2.3% (for pulp yield), and the minimum percentage of phenotypic variance explained by a significantly associated SNP was 0.33% (CBH age 3 yr).

### 2.4 GWAS for dominance effects

**FIGURE 3:**
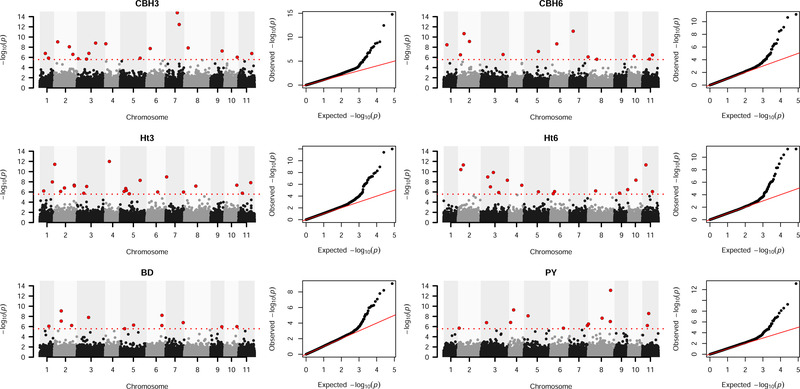
Manhattan plots and quantile‐quantile (QQ) plots of the fixed and random model circulating probability unification (FarmCPU) results for the dominance effects model. The traits used are circumference at breast height and height at age 3 and 6 (Ht3 and Ht6, respectively) as well as basic density (BD) and pulp yield (PY). The Manhattan plots show –log_10_ *p* values plotted against SNP positions on the 11 *Eucalyptus* chromosomes. Associations reaching genome‐wide significance are displayed in red and the horizontal dotted line indicates a Bonferroni‐corrected significant threshold of *p* < 1.7 × 10^–6^. The QQ plots for each of the six traits indicate the observed versus expected distribution of *p* values. The solid red line represents the expected null distribution assuming no associations. CBH3, circumference at breast height at age 3 yr; CBH6, circumference at breast height at age 6 yr

The FarmCPU efficiently controlled the false‐positive rates due to population structure and sample relationships when identifying significant loci using dominance encoding (Figure 3, QQ plots). Under a dominance model, we detected a total of 82 significant SNPs for the six traits. Height at 3 yr old (Ht3) had the greatest number of associations, with 19 SNPs displaying significant effects. Fewer associations were observed for the other traits, with between 11 and 15 significant SNPs identified (Figure 3, Manhattan plots). Two significant SNPs, Chr5.40663824 and Chr11.28479550, were found to overlap between CBH and Ht6. The maximum percentage of phenotypic variance explained by an associated SNP was 4%, a much higher value than found in the additive effect estimations. The smallest percentage of phenotypic variance explained by an associated SNP for the dominance model was of similar magnitude to that observed for additive effects model (Table S2). Comparing significant SNPs identified from the additive and dominance effects models, a total of 10 SNPs overlap between two models for different traits. This result suggests that the two genetic effects are not entirely independent. Nine out of 10 SNPs that overlap between additive and dominance effects were identified for growth traits, with the remaining SNP observed for pulp yield.

### 2.5 Genome selection by using GWAS results

To confirm the utility of the SNPs identified from the GWAS and to further understand the performance of selecting SNPs for each trait based on the GWAS results, we conducted genomic prediction using four categories of SNPs based on the results from the GWAS analyses. The four categories of SNPs used for the genomic best linear unbiased prediction analyses were selected from the GWAS results for each trait based on the following criteria: (a) associated SNPs were identified as significant from the GWAS using the threshold *p *< 1.7 × 10^–6^; (b) putative SNPs were identified as significant from the GWAS using a more relaxed significance threshold *p *< 1 × 10^–3^ of each trait; (c) the top 1% SNPs included the top 1% SNPs for each trait, ranked according to GWAS significance. The rationale of this category was to ensure that models utilized the same number of SNPs across all traits. Finally, (d) all SNPs used all 37,832 available SNPs when building the genomic selection models (Table S4).

Using markers identified from a GWAS for subsequent GS in the same population will inflate prediction accuracies as the GWAS and GS results are not independent (McGowan et al., 2021). To circumvent the issue of non‐independence in the evaluation of GS prediction accuracies, we used a cross‐validation approach where the complete data set was split into a discovery and training population consisting of 90% of the individuals and a validation population of the remaining 10% of individuals. The allocation of individuals to discovery and training set or validation set was done randomly. The GWAS was then re‐run only in the discovery and training population, while the accuracy of the GS model was evaluated only in the validation population. The separation of GWAS discovery and GS validation to separate subsets of the original population assures that the results will not be inflated due to non‐independence. The SNPs from the four different categories outlined above were used to calculate G‐matrices for either additive or dominance effects and then used to predict either breeding values based on the additive G matrix (the A model) or total genetic values based both additive and dominance G matrices (the AD model). Prediction accuracy in the validation set was estimated as the correlation between observed phenotypes and predicted breeding values for the A model (Figure 5) or the correlation between observed phenotypes and predicted genetic values for the AD model (Figure 5). For each trait and SNP category, the process described above was repeated 100 times to assess variation in prediction accuracy. Similarly, narrow‐sense and broad‐sense heritabilities were estimated based on the different maker‐based relationship matrices calculated for the four SNP categories.

**FIGURE 4:**
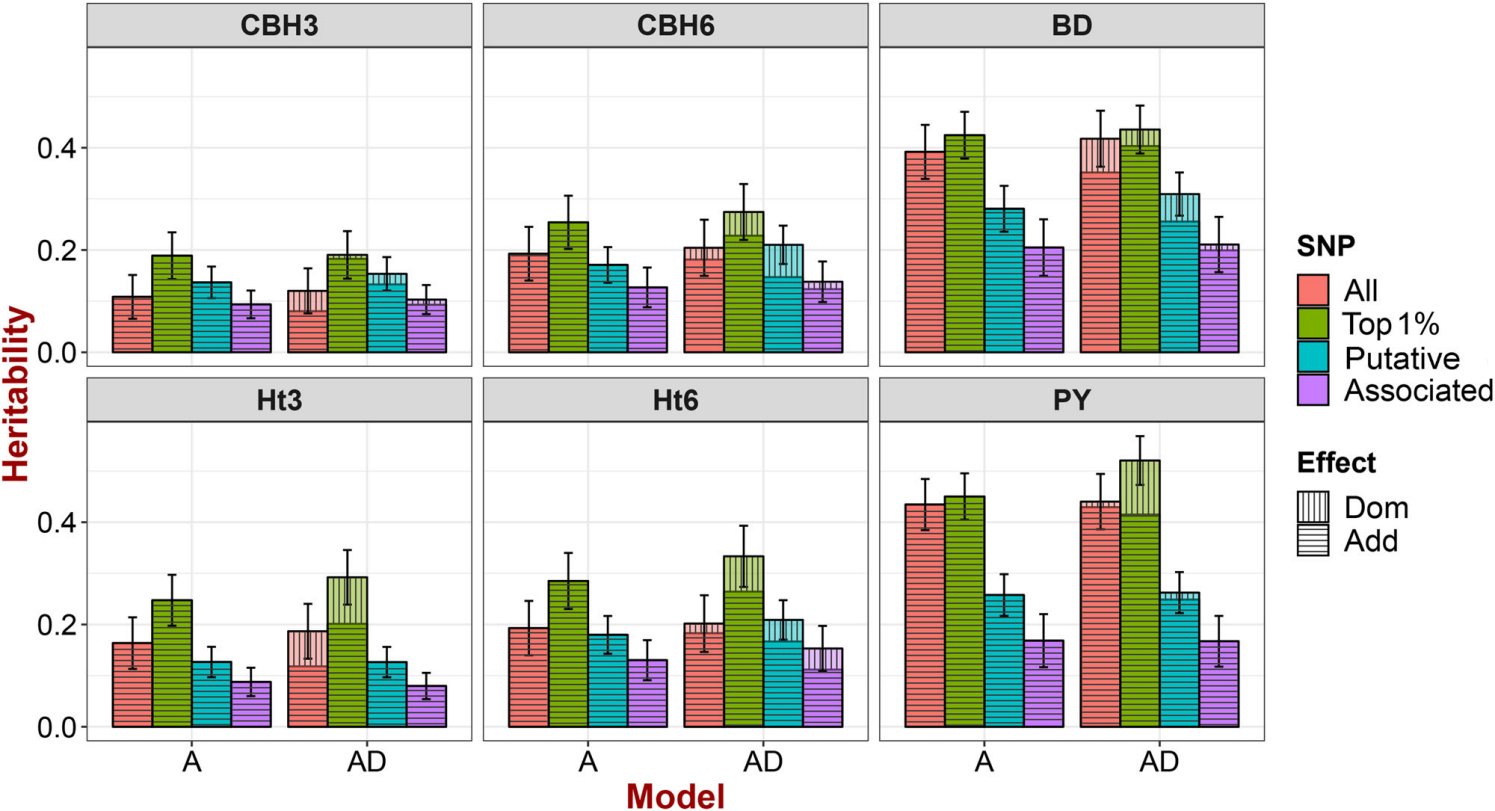
Genomic‐based narrow‐ and broad‐sense heritabilities based on an additive (*A*) or an additive + dominance (*AD*) model for the four different categories of single nucleotide polymorphisms (SNPs) used. The colored bins represent the different categories of SNPs used, with red indicating all SNPs (37,832), green indicates the top 1% SNPs ranked according to genome‐wide association studies (GWAS) *p* value, cyan denotes putative SNPs selected based on GWAS results with *p* < 10^–3^ and purple denoted associated SNPs selected based on GWAS results using *p* < 1.7 × 10^–6^. The fill patterns represent different genetic effects. Vertical lines denote additive effects and horizontal lines denote dominance effects. Error bars indicate the standard error of total genetic variance. Add, additive effect; BD, basic density; CBH3, circumference at breast height at age 3 yr; CBH6, circumference at breast height at age 6 yr; Dom, dominance effect; Ht3, height at age 3 yr; Ht6, height at age 6 yr; PY, pulp yield

As expected, basic density and pulp yield had higher realized heritabilites than growth traits, independent of what category of SNPs were used for the calculations. Broad‐sense heritabilities were higher than narrow‐sense heritabilities for most traits, demonstrating that dominance plays an essential role in the expression of most traits (Figure 4) and in line with earlier observations in this population (Tan et al., 2018). Comparing heritabilites (*h* ^2^ and *H* ^2^) for the different SNP categories suggest, perhaps surprisingly, that the top 1% SNPs category explain more of the genetic variation than any of the other categories, including when all SNPs were used (Table S4). Furthermore, using the top 1% SNP set yielded the largest estimates of dominance effects. As expected, using only SNPs that were significantly associated with a trait in the GWAS resulted in lower heritability estimates than using all SNPs. Comparing heritability estimates between the putative and all SNP categories showed that these yielded similar estimates for CBH and height. The putative category of SNPs yields significantly lower heritability estimates than the all SNP category for the two wood quality traits (Figure 4).

**FIGURE 5:**
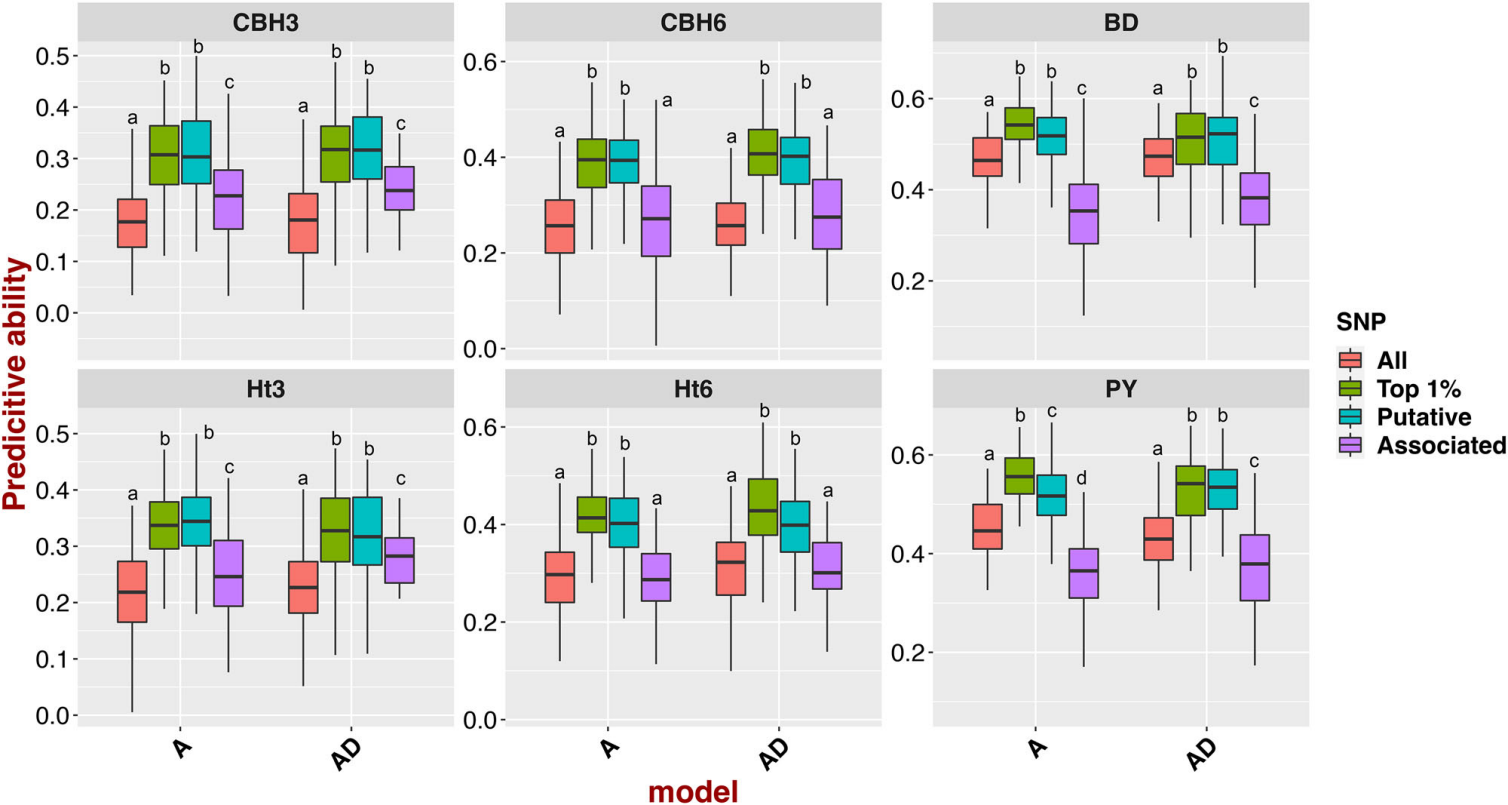
Predictive abilities of the additive (A) and the additive + dominance (AD) genomic prediction models for the four different categories of SNPs. The colored boxplots display the distribution of predictive ability across 100 replicates of 10‐fold cross‐validation for the different categories of SNPs. Colors used as in Figure 4. Groups with different letters (a–d) are significantly different based on a Tukey honestly significant difference test. BD, basic density; CBH3, circumference at breast height at age 3 yr; CBH6, circumference at breast height at age 6 yr; Ht3, height at age 3 yr; Ht6, height at age 6 yr; PY, pulp yield

We further estimated the prediction ability of breeding values for the *A* model and the prediction ability of genetic values for the *AD* model using a using cross‐validation approach. The distribution of predictive abilities for each of the models and SNP, obtained using 100 replications, are displayed in Figure 5. Generally, we observe higher prediction abilities for wood quality traits, in line with the higher heritability values we observe for these traits. The AD model yielded slightly higher prediction abilities than the A model for most traits, but these differences are not significant. When comparing the different SNP categories, both the top 1% and putative SNP categories yielded substantial improvements in the predictive ability compared with both the all and the significant SNP categories, and this was consistent across the A and AD models. The associated SNPs yielded more or less similar results to those obtained using all SNPs. Moreover, the top 1% SNP category yielded the highest average prediction ability for height, basic density, and pulp yield, with values being much higher than those calculated from the all SNP category (Table S6).

## 3 DISCUSSION

We used GWAS with both additive and dominance effects to dissect the genetic architecture of growth and wood quality traits in hybrid *Eucalyptus*. The method we used for GWAS, FarmCPU, was able to control the false‐positive rate induced by both the complex population structure and kinship that characterize our mapping population and efficiently identified significantly associated SNPs for both additive and dominance effects. Using top‐ranking SNPs based on the GWAS yielded higher genomic heritability estimates than using all available SNPs. We were also able to achieve more accurate genomic prediction results by filtering SNPs based on their associations in the GWAS, and this helped shed light on future directions in the application of genomic selection in *Eucalyptus* breeding.

### 3.1 FarmCPU have superior performance in GWAS analyses

Most economic traits targeted for breeding in forestry, such as growth and wood properties, are quantitative traits and are thought to have a complex genetic architecture controlled by many loci of small effect. Here we utilized a recently developed method for the dissection of complex traits, FarmCPU, that has been proposed to efficiently address problems confounding between testing markers and covariates that often arise in GWAS (Liu et al., 2016). Several empirical studies have verified that FarmCPU offers enhanced power for GWAS of complex traits (Vanous et al., 2019; Ward et al., 2019; Zhu et al., 2018). In this study, we identified 78 and 82 significant associations having additive and dominant effects, respectively, with an average of 13 SNPs identified per trait studied. These results are more efficient when compared with other commonly used GWAS methods, such as the genome‐wide efficient mixed model association (GEMMA). In preliminary analyses, we found that GEMMA was also able to control the false‐positive rate very well, but this came at the price of low statistical power. GEMMA consequently only identified two significant SNPs for additive effects (Figure S4) and a total of 13 significant SNPs for the dominance effects across traits (Figure S5). The FarmCPU methods, therefore, appear to be an attractive method that strikes a good balance between the identification of false positives and false negatives and which yields good power for the dissection of complex traits.

### 3.2 Dominance effects play important roles in hybrid population

GWAS have traditionally assumed only additive effects of individual SNPs (Bush & Moore, 2012; Marjoram et al., 2014), but here we show the added value of also considering dominance effects for identifying genomic regions controlling growth and wood quality traits. By assessing dominance effects, we identify an additional 72 associated SNPs across the traits in addition to the 78 SNPs we identified using additive effects. Furthermore, a considerable proportion of the genetic variation in our hybrid population is attributable to non‐additive effects, and our results show that the alleles underlying this variation can be identified when dominance effects are explicitly considered in a GWAS setting. Several previous studies have used controlled crosses in crop species, particularly in maize and rice, to identify loci that exhibit dominance effects. For heterosis‐related traits, data from maize is frequently cited as supporting the dominance model (Cui et al., 2017; Wallace et al., 2014). Rice, on the other hand, has been proposed as a system that supports the over‐dominance hypothesis (Li et al., 2016; Zhen et al., 2017). Our approach quantifies the contribution of dominance to the “missing heritability” in a *Eucalyptus* hybrid breeding population, and we collectively show that up to 10% of the genomic‐based heritability can be explained by associated SNPs that were identified using a dominance model (Table S5).

### 3.3 The benefit of integrating GWAS results on genomic prediction

Even if we capture a substantially larger number of associated SNPs by considering both additive and dominance effects, a large fraction of the genomic‐based heritabilities (14−62%) cannot be explained by only considering significantly associated SNPs (Table S5), and these observations are in line with several earlier reports (Chhetri et al., 2019; Tang et al., 2019; Zhao et al., 2019). Also, when using significantly associated SNPs from the GWAS to investigate the accuracy of genomic prediction, we find that this yields no improvement in accuracy, and sometimes even reduced accuracy, compared with predictions based on all available SNPs, which mirrors results seen in other similar studies (Gowda et al., 2015; Wallace et al., 2016). Regions identified in a GWAS are consequently not able to explain all genetic variation in the traits of interest, and this problem is greater for quantitative traits that are controlled by many genes of small effect. These are the traits where current GWAS methods often suffers from insufficient power to detect loci of small effect unless sample sizes are substantially larger than what is commonly used in most studies of plants.

To assess if the GWAS results could be used to enhance genomic prediction in our breeding population, we also tried to identify possible candidate SNPs that were not detected as significant using the stringent significant threshold we applied in our GWAS. The rationale here is that, as outlined above, most GWAS methods fail to detect loci of small effect but that the GWAS would nevertheless serve as a useful filter for ranking SNPs for their possible effects on the traits of interest. We, therefore, selected two categories of SNPs using two different criteria of relaxed significance and used these to estimate genomic heritabilities and perform genomic prediction. The first category, putative SNPs, includes all SNPs that were found to be associated with the traits of interest based a more relaxed *p* value (*p *< 1 × 10^–3^). Using this more relaxed *p* value, we identify between 70 to 184 SNPs for the different traits when combined across the additive and dominance effect models. Using the putative SNP category, we observed large improvements in the heritability estimates for the growth traits, to the point where almost all of the genetic variation could be explained (Table S5). For wood quality traits, however, about 40% of the genetic variation remain unexplained compared with when using all SNPs for heritability estimation (Table S5). The second category of SNPs we considered consisted of the top 1% of SNPs, ranked by the *p* value from the GWAS. Using this criterion ensures that the same number of SNPs are used for prediction across the different traits. Surprisingly we were able to explain a substantially greater proportion, up to 174%, of the genetic variation explained when using all SNPs (Table S5). When we performed genomic prediction using these two categories of SNPs, we also observed a substantial increase in the prediction ability for all traits compared with predictions based on all available SNPs. This suggests that using all available SNPs introduce noise in the prediction models that negatively affects our prediction ability. Our method for analyzing genomic selection and increasing prediction accuracy benefited from integrating results from the GWAS analyses, but the number of associated SNPs that need to be incorporated depends on the trait in question.

We have relied on a cross‐validation approach to ensure that the GS prediction results are not inflated due to non‐independence between the GWAS and GS population. However, a better approach would be to separate the GWAS discovery population completely from the GS model building population, although this may be difficult for logistical reasons in many species, and especially in forest trees like *Eucalyptus* where field trials can cover several hectares or more.

### 3.4 Detection of associations for complex traits in forest trees

Identifying candidate genes underlying growth and wood traits has long been an active area of research in forest trees, such as in *Eucalyptus* (Cappa et al., 2013; Müller et al., 2017; Müller et al., 2019; Resende et al., 2017a), *Populus* (Allwright et al., 2016; Du et al., 2016; Fahrenkrog et al., 2017; Porth et al., 2013), and *Pinus taeda L*. (Bartholomé et al., 2016; Lu et al., 2017). To ensure good statistical power, both common and rare genetic variants need to be considered to gain a comprehensive understanding of the genetic regulation of complex traits since many low‐frequency variants were identified as associated with growth and wood composition traits (Fahrenkrog et al., 2017). For instance, regional heritability mapping (RHM) has previously been shown to successfully utilize information from both common and rare variants and can therefore capture a larger proportion of the genomic heritability in *Eucalyptus* (Müller et al., 2019; Resende et al., 2017a).

Furthermore, both additive and non‐additive effects play important roles in association studies for many traits. Adding dominance effects to a GWAS analysis increase the possibility to identify additional variants that can help capture a greater fraction of the genetic variance (Du et al., 2016; Lu et al., 2017). Other methods, such as increasing the sample size using meta‐analysis (Müller et al., 2019) or using multi‐locus GWAS approaches instead of single‐marker methods (Fahrenkrog et al., 2017) are methods that also can help increase statistical power in GWAS.

### 3.5 Putative genes for plant growth and stress response

Among the significantly associated SNPs we observe across additive and dominance effect estimations, we identified a total of 49 candidate genes that have known functions relevant for the traits in question. The details of these genes, including information on the position of associated SNPs and the putative functions of the genes, are summarized in Table S7. In general, candidate genes can be separated into two groups, with one group containing genes that have direct functions associated with the morphological formation of different tissues or organs. The other group contains genes related to general responses to abiotic and biotic stress, which, more indirectly, influence plant growth and biomass.

Among the significant SNPs associated with morphology, a number of associations are linked to genes that are related to cell wall biosynthesis. For example, SNP Chr3.46653967 is associated with Ht6 using an additive effects model. This SNP is located on chromosome 3 and encodes a missense variant in a gene coding for a pectin lyase‐like superfamily protein (*PME*). This gene is expressed in stamen and is involved in cell wall loosening and has previously been implicated in floral development (Francis et al., 2006). We also identified a significant SNP on chromosome 11 (Chr11.20479646), which is associated with Ht6 (Table S7). This SNP is located in the gene *Eucgr.K01691*, which encodes a homolog to the Arabidopsis alpha‐L‐arabinofuranosidase 1 (*ARAF*1) gene. Expression of the *ARAF*1 gene is localized to several cell types in the vascular system of roots and stems, and the protein is known to be involved in cell‐type‐specific alterations of cell wall structure (Chávez Montes et al., 2008). Many other studies have also identified cell wall biosynthesis‐related genes from GWAS, performed using growth traits in forest trees. Du et al. (2016) identified four significant SNPs located in genes involved in secondary cell wall biosynthesis when analyzing growth traits in the *Populus* (Du et al., 2016). A SNP associated with volume in *Eucalyptus pellita* F. Muell. is located in a gene whose function is known to be involved in cell wall cellulose biosynthesis (Müller et al., 2017). Similarly, Müller et al. (2019) used a joint‐GWAS approach in four *Eucalyptus* breeding populations and identified eight SNPs associated with growth traits that were all linked to genes that were related to cell wall biosynthesis.

Many of the candidate genes putatively related to abiotic and biotic stress show responses to adverse conditions. It is perhaps not surprising that these genes show up in our GWAS, as the planting area of the study population alternates between extremely dry (from July to August) and wet (from August to October) conditions in most years, which often leads to stress‐induced damage and high incidence of diseases. In line with this, we identified several candidate genes involved in stress response to adverse climate conditions. The SNP Chr2.1760161 is highly associated (*p* value = 3.72 × 10^–12^) with Ht3 in the dominance model. This SNP is located upstream of the gene *Eucgr.B00092*, which encodes a putative *HVA*22 homologue E (*HVA*22*E*). *HVA*22*E* is upregulated to varying degrees in response to cold and salt stress, ABA treatment or dehydration (Chen et al., 2002; Shen et al., 2001). Another variant (Chr3.41941452), associated with CBH6, is located in the upstream region of *High‐affinity K+ transporter* 1 (*HKT*1) gene. *HKT*1 is expressed in root stelar cells and leaf cells (Hamamoto et al., 2015) and provides a key mechanism for protecting leaves from Na+ over‐accumulation and salt stress (Berthomieu et al., 2003; Maser et al., 2002). The SNP Chr6.23066996 is associated with CBH6 in the additive effects model and is located on chromosome 6 inside the gene *Eucgr.F01775* that encodes catalase 2 (*CAT*2). *CAT*2 controls levels and sensitivity to H_2_O_2_ (Bueso et al., 2007), photo‐oxidative stress (Konert et al., 2015) and auxin levels (Gao et al., 2014).

Four of the candidate genes we identify in our GWAS have functions in both morphological formation and stress response. One common SNP (Chr11. 28479550), associated with CBH6 in both the additive and dominance models as well as with Ht6 for the dominance model, is located in the vicinity of the gene *Eucgr*.K02133, which encodes a nucleotide‐diphospho‐sugar transferase (*QUA*1). This enzyme is expressed in vascular tissues and affects homogalacturonan, pectin, and hemicellulose cell wall synthesis (Orfila et al., 2005). Recent studies have shown that *QUA*1 also functions in chloroplast‐dependent calcium signaling under salt and drought stresses (Zheng et al., 2016). Finally, the SNP Chr4.11644680 is associated with Ht3 and is a synonymous variant located in the *SFR*6/*MED*16 gene, which plays important roles in cold‐ and drought‐inducible gene expression (Knight et al., 2009), defense gene expression (Wathugala, 2012), as well as modulating iron uptake (Zhang et al., 2014) in response to cell wall defects (Sorek et al., 2015). These findings suggest that stress resistance also plays an important role in affecting tree growth traits.

## 4 CONCLUSIONS

In this study, we have used a GWAS approach in a *Eucalyptus* hybrid population to dissect the genetic basis of growth and wood quality traits by accounting for both additive and dominance genetic effects. Altogether we identified 78 and 82 significant SNPs using additive and dominance models, respectively, with 10 SNPs showing overlap between the two effect models, suggesting that additive and dominance effects are not completely independent. The associated genes could be grouped into two broad functional categories relating to how they influence tree growth and biomass. One group contains genes associated with morphological formation, such as cell wall biosynthesis, and the other group contains genes related to abiotic and biotic stress responses, such as oxidative, hormone‐based, and disease‐induced stress. These results provide novel targets for possible transgenic or genome editing approaches in the future to directly improve growth and biomass related traits.

We also applied our results from the GWAS in a genomic selection analysis by using different categories of SNPs selected based on the GWAS results and used them to evaluate genomic‐based heritabilities and predictive abilities. Our results show that prediction abilities of the estimated breeding values improved for all traits when using SNPs selected based on the GWAS results. Integrating GWAS results into genomic selection thus appears to be a promising avenue to increase the efficiency of genomic selection in forest breeding.

### 4.1 Experimental procedures

#### 4.1.1 Populations, phenotypic and genotypic data

A total of 1,123 *Eucalyptus* individuals were used in this study, including 90 *Eucalyptus grandis*, 84 *Eucalyptus urophylla* parents and 949 F1 progenies derived from a random mating design that has previously been described (Tan et al., 2017). Briefly, F1 individuals were identified to be comprised of inter‐ and intra‐crossing of the two parental species. Of the 949 F1 individuals, 57% were interspecific *E. grandis* × *E. urophylla* hybrids, 21% were intraspecific *E. grandis* × *E. grandis* progeny, and 22% were intraspecific *E. urophylla* × *E. urophylla* progeny (Tan et al., 2018).

The phenotypic and genotypic data utilized in this study has been previously described in detail (Tan et al., 2017). The phenotypes include height and CBH, where F1 individuals were evaluated at ages 3 and 6 and the pure species parents were evaluated at age 5. In addition, we obtained data on two wood quality traits, basic density and pulp yield, that were assessed at age 5. Genotyping was performed using an Illumina Infinium EuCHIP60K SNP chip that contains probes for 60,904 unique SNPs (Silva‐Junior et al., 2015). Across the 1,123 individuals, 37,832 SNPs were retained after quality‐control based on call rates (>0.7) for both SNPs and samples and following filtering based on minor allele frequencies (>0.01) and deviations from Hardy‐Weinberg equilibrium (>10^–7^). Any missing data remaining in the 37,832 SNPs were subsequently imputed using BEAGLE 4.1 (Browning & Browning, 2007).

#### 4.1.2 Phenotypic data analyses

Phenotype data for the parental and F1 population were adjusted separately to minimize environmental variation by fitting a mixed linear model for each trait:

(1) $$y=X\beta +Z_{b}r+\varepsilon$$

where **y** is the vector of phenotypic observation, **β** is the vector of overall mean as a fixed effect, **r** is the vector of random replication effects following $r∼N(0,\;I\sigma_{r}^{2})$, where $\sigma_{r}^{2}$ is the replication variance, **ε** is the vector of random residual effects. **X** and **Z_b_** is design matrix for **β** and **r**, respectively. For the F1 population, the residual variance‐covariance matrix is $R\;=\;{I\sigma }_{e}^{2}+\mathrm{AR}1\left(\rho_{r}\right)⊗\mathrm{AR}1\left(\rho_{c}\right)\sigma_{\eta }^{2}$, where $\mathrm{AR}1(\rho_{r})$ and $\mathrm{AR}1(\rho_{c})$ are autoregressive correlation matrices for the row model (autocorrelation parameter ρ*_r_*) and column model (autocorrelation parameter ρ*_c_*), respectively. $\sigma_{e}^{2}$ is the independent residual variance, while $\sigma_{\eta }^{2}$ is the spatial variance. For the pure parental species, we fitted the model in Equation 1 by setting the residual matrix $R\;=\;I\sigma_{\varepsilon }^{2}$ since spatial coordinates and position information were not available for these individuals. All mixed‐linear model analyses were performed in ASReml 4 (Gilmour et al., 2015). Phenotypes of individuals from the F1 and parental populations were adjusted for random block effects (*r*) and spatial effects (*s*), respectively.

The heritability (*h* ^2^) was estimated using a mixed model *y* = X**β** + **Zu** + ε, where y represents the adjusted phenotypes of a single trait, **β** is the vector of fixed effects, including overall mean and age difference. Also, **u** is a vector of random additive or dominance genetic effect of individuals with a normal distribution, u∼N(0, A$\sigma_{a}^{2}$), A being the revised, pedigree‐based genetic relationships among individuals (Tan et al., 2018); and ε is a heterogeneous random residual effect representing different experimental sites. **X** and **Z** are incidence matrices for β and *u*, respectively. We obtained restricted maximum likelihood estimates of $\sigma_{a}^{2}$ and ε and estimated $\;h^{2}=\sigma_{a}^{2}\;/\left(\sigma_{a}^{2}+\frac{\varepsilon }{n}\right)$.

### 4.2 Population structure, kinship analysis and GWAS

In the association analyses, confounding effects of population structure and kinship between individuals need to be accounted for. Population structure (Q) was estimated using a model‐based clustering and through PCA using 13,245 independent SNPs obtained by LD‐pruning the original SNP data set and including only SNPs that have pairwise linkage disequilibrium (LD) values (*r* ^2^) less than 0.2. Model‐based clustering was implemented using the software *admixture* v.1.3.0, which infer population structure by estimating individual admixture proportions using multi‐locus SNP data through a maximum‐likelihood method (Alexander et al., 2009). The number of ancestral populations (P) was varied from 1 to 100 when using admixture and *fastStructure* (Raj et al., 2014). Five‐fold CV was performed to choose the optimal *p* value in admixture. A PCA was also performed using the *smartpca* program in *eigensoft* v6.0 to estimate individual ancestry proportions (Price et al., 2006).

A GWAS was conducted using a recently developed method, FarmCPU, which explicitly takes into account the confounding that exists between covariates and test marker using both a fixed effect model and a random effect model (Liu et al., 2016). The results from the PCA and the kinship matrix were used as covariates in FarmCPU to account for population structure and relatedness among samples, respectively. We ran the GWAS using the R package *FarmCPU*. False‐positive errors due to multiple testing were controlled by an adjusted Bonferroni method, *simpleM* (Gao et al., 2008). This method infers the number of independent SNPs by filtering on LD and performs a standard Bonferroni correction to correct for multiple testing based on the number of independent tests performed. For the present data, a *p* value < 1.7 × 10^–6^ was selected as a cut‐off to indicate significant associations.

### 4.3 GWAS for additive and dominance models

We conducted genome‐wide association analyses in FarmCPU using either an additive or dominance encoding of genotypes. For the additive encoded data, the homozygous major allele was encoded with 0, the heterozygous genotype with 1, and the homozygous minor allele with 2. For the dominance encoding, both homozygous minor and major alleles were encoded as 0, whereas the heterozygous genotype was encoded as 1 (Seymour et al., 2016).

### 4.4 Genomic selection with different informative SNPs

Genomic selection models were constructed based on four different categories of SNPs using the genomic best linear unbiased prediction method. Details on how the genomic‐based additive and dominance relationship matrices are estimated have been previously described in detail in Tan et al. (2018).

Here we focus on the details of how the four categories of SNPs we have used in all subsequent analyses were selected. In all analyses, the entire population was split into two parts by randomly allocating individuals to one of two possible sub‐populations where 90% of the individuals make up the discovery and training population and the remaining 10% make up a validation population. The discovery and training population was used to run GWAS using FarmCPU to identify *p* values for all SNPs assuming additive and dominance effects, respectively. The *p* values were then ranked from the lowest to highest for each trait that was assessed, and the SNPs were used to set up four non‐mutually exclusive categories based on the GWAS results. The first SNP category contains associated SNPs and includes all SNPs that are significantly associated with a trait at a *p* value < 1.7 × 10^–6^. The second SNP category, putative, includes all SNPs that are associated with a particular trait with *p* values < 10^–3^ and thus also includes SNPs that are putatively associated with the trait but that does not reach proper statistical significance in the GWAS. The third SNP category, top 1%, select the top 378 SNPs based on their *p* values to be able to compare similar‐sized SNP sets across traits despite having very different *p* value distributes. Finally, the fourth SNP category, all, includes all SNPs and is equivalent to the standard GS procedure where SNPs are not subject to filtering, which therefore is identical to the models originally used in Tan et al. (2017) and Tan et al. (2018).

The four SNP categories were then used to separately calculate G matrices based on either additive or dominance effects from the discovery and training set and used to predict breeding values in the validation set using either only the additive G matrix (the *A* model) or using both additive and dominance G matrices to predict the total genetic value for the validation set (the *AD* model). The *A* and *AD* models have been well described earlier in Tan et al. (2018). The genomic‐based narrow‐ and broad‐sense heritability (*h* ^2^ and *H* ^2^, respectively) were calculated after fitting each model across the different traits. Narrow‐sense heritability in the *A* model was estimated as $h^{2}=\sigma_{a}^{2}\;/\sigma_{p}^{2}$ and the broad‐sense heritability of *AD* model was estimated as $h^{2}=\;\left(\sigma_{a}^{2}+\sigma_{d}^{2}\right)/\sigma_{p}^{2}$, where $\sigma_{a}^{2},\;\sigma_{d}^{2}$and $\sigma_{p}^{2}$ represented the estimated additive, dominance, and phenotypic variance, respectively. For the validation set, we calculated correlations between observed phenotypes and predicted breeding values, *r*(*Â_vali_*, *Y_vali_*), or correlations between observed phenotypes and predicted genetic values, *r*(*Ĝ_vali_*, *Y_vali_*). For each trait and SNP category, the process described above was repeated 100 times to assess variation in prediction accuracy. To assess differences in prediction accuracy between the four SNP categories, we used a one‐way analysis of variance followed by a Tukey honestly significant difference post‐hoc test.

### 4.5 Assigning significant SNPs to putative candidate genes

Genes within ±5 kb away from a SNP that was significantly associated with a measured phenotypic trait were extracted from the *E. grandis* v2.0 reference genome (BRASUZ1) in Phytozome (www.phytozome.net) using SnpEff v4.2 (Cingolani et al., 2012). The 5‐kb window threshold used was based on the distance over which LD decays in this population (Tan et al., 2017). The putative functions of these candidate genes were determined based on their homology to functionally characterized genes in *A. thaliana* (TAIR).

## AUTHOR CONTRIBUTIONS

Biyue Tan: Data curation; Formal analysis; Investigation; Methodology; Validation; Visualization; Writing – original draft; Writing – review & editing. Pär K. Ingvarsson: Conceptualization; Data curation; Funding acquisition; Project administration; Resources; Supervision; Visualization; Writing – review & editing.

## CONFLICT OF INTEREST

Biyue Tan is employed by Stora Enso AB. Pär K. Ingvarsson reports no conflict of interests.

## Supporting information


**Figure S1**. The distribution and correlation of phenotypes. Scatter plot (lower off‐diagonal) and correlations with probability values (upper off‐diagonal; H_0_: r = 0) for adjusted phenotype among the study traits. Diagonal is the histograms of the distribution of the study phenotypes.
**Figure S2**. The cross‐validation error of admixture analysis. K in x‐axis represent the test of clustering the population of parents and F1 progenies into the number of groups. Here we tested K from 1–100. The smaller in CV_error means the number of the cluster is the best.
**Figure S3**. The eigenvalue of the principle component analysis for testing population structure. *X* axis represent the number of principle component were used and the eigenvalue of each component.
**Figure S4**. Manhattan plots and quantile‐quantile (QQ) plots represent the GEMMA results of estimating additive genetic effects. CBH and height (Ht) at 3 years and 6 years, respectively, basic density (BD) and pulp yield (PY) traits are participated the analyses. Manhattan plots indicate negative log10 *p* values from a genome‐wide scan are plotted against SNP positions of 11 chromosomes. The horizontal red lines indicate the significant threshold of *p* value < 1.7 × 10^–6^. QQ plots of each trait showing expected null distribution of *p* value vs. distribution of observed *p* value, the solid red line represents the null distribution assuming no associations.
**Figure S5**. Manhattan plots and quantile‐quantile (QQ) plots represent the GEMMA results of estimating over‐dominance genetic effects. CBH and height (Ht) at 3 years and 6 years, respectively, basic density (BD) and pulp yield (PY) traits are participated the analyses. Manhattan plots indicate negative log10 *p* values from a genome‐wide scan are plotted against SNP positions of 11 chromosomes. The horizontal red lines indicate the significant threshold of *p* value < 1.7 × 10^–6^. QQ plots of each trait showing expected null distribution of *p* value vs. distribution of observed *p* value, the solid red line represents the null distribution assuming no associations.

Supplementary Table Legends
**Table S1**. Associated significant SNPs estimated by additive genetic effect GWAS model
**Table S2**. Associated significant SNPs estimated by dominance genetic effect GWAS model
**Table S3**. Summary of significant GWAS associations for A) additive and B) dominance effects.
**Table S4**. Summary of the number of SNPs with different filter type
**Table S5**. Summary of heritability for model estimated by different type of SNPs
**Table S6**. Mean and standard deviation for predictive ability of the two genetic models for the different categories of SNPs
**Table S7**. Candidate genes identified according to significant SNPs for different model
